# Correction: Tuning the charge carrier mobility in few-layer PtSe_2_ films by Se : Pt ratio

**DOI:** 10.1039/d1ra90174e

**Published:** 2021-12-08

**Authors:** Jana Hrdá, Valéria Tašková, Tatiana Vojteková, Lenka Pribusová Slušná, Edmund Dobročka, Igor Píš, Federica Bondino, Martin Hulman, Michaela Sojková

**Affiliations:** Institute of Electrical Engineering, SAS Dúbravská cesta 9 84104 Bratislava Slovakia michaela.sojkova@savba.sk; Faculty of Electrical Engineering and Information Technology, Slovak University of Technology Ilkovičova 3 81209 Bratislava Slovakia; Faculty of Mathematics, Physics and Informatics, Comenius University in Bratislava Mlynská dolina F2 84248 Bratislava Slovak Republic; IOM-CNR, Laboratorio TASC S.S. 14 km 163.5, 34149 Basovizza Trieste Italy

## Abstract

Correction for ‘Tuning the charge carrier mobility in few-layer PtSe_2_ films by Se : Pt ratio’ by Jana Hrdá *et al.*, *RSC Adv.*, 2021, **11**, 27292–27297, DOI: 10.1039/D1RA04507E.

The authors regret the omission of a funding acknowledgement in the original article. This acknowledgement is given below.

The research leading to this result has been supported by the project CALIPSOplus under Grant Agreement 730872 from the EU Framework Programme for Research and Innovation HORIZON 2020.

The Royal Society of Chemistry apologises for these errors and any consequent inconvenience to authors and readers.

## Supplementary Material

